# Ability of Constitutive Models to Characterize the Temperature Dependence of Rubber Hyperelasticity and to Predict the Stress-Strain Behavior of Filled Rubber under Different Defor Mation States

**DOI:** 10.3390/polym13030369

**Published:** 2021-01-25

**Authors:** Xintao Fu, Zepeng Wang, Lianxiang Ma

**Affiliations:** College of Electromechanical Engineering, Qingdao University of Science and Technology, Qingdao 266061, China; fuxintao_qust@163.com

**Keywords:** hyperelasticity, constitutive model, temperature dependence, finite element analysis, filled rubber

## Abstract

In this paper, some representative hyperelastic constitutive models of rubber materials were reviewed from the perspectives of molecular chain network statistical mechanics and continuum mechanics. Based on the advantages of existing models, an improved constitutive model was developed, and the stress–strain relationship was derived. Uniaxial tensile tests were performed on two types of filled tire compounds at different temperatures. The physical phenomena related to rubber deformation were analyzed, and the temperature dependence of the mechanical behavior of filled rubber in a larger deformation range (150% strain) was revealed from multiple angles. Based on the experimental data, the ability of several models to describe the stress–strain mechanical response of carbon black filled compound was studied, and the application limitations of some constitutive models were revealed. Combined with the experimental data, the ability of Yeoh model, Ogden model (*n* = 3), and improved eight-chain model to characterize the temperature dependence was studied, and the laws of temperature dependence of their parameters were revealed. By fitting the uniaxial tensile test data and comparing it with the Yeoh model, the improved eight-chain model was proved to have a better ability to predict the hyperelastic behavior of rubber materials under different deformation states. Finally, the improved eight-chain model was successfully applied to finite element analysis (FEA) and compared with the experimental data. It was found that the improved eight-chain model can accurately describe the stress–strain characteristics of filled rubber.

## 1. Introduction

The accuracy of the hyperelastic constitutive model of rubber materials has an important influence on the mechanical analysis and calculation accuracy of rubber structure. The mechanical properties of rubber can be significantly affected by the change of temperature [1]. Therefore, it is particularly important to screen or propose models that can accurately characterize the mechanical properties of rubber at different temperatures and can be easily applied to the finite element analysis software. So far, the understanding and characterization of the mechanical behavior of rubber materials have been greatly developed. At the same time, many constitutive models have been developed for rubber materials and a lot of experimental data have been obtained [2,3]. Different hyperelastic models have different capabilities to predict the complex deformation behavior of rubber materials. Seibert et al. discussed the predictive ability of six hyperelastic models to test data [4]. Steinmann et al. investigated the fitting ability of 14 hyperelastic models to test data under different deformation states [5]. Hohenberger et al. studied the constitutive model for both low and high strain non-linearities in highly filled elastomers and implementation [6]. Ding made a theoretical summary on how to choose the model based on the analysis of uniaxial stress–strain curves [7]. However, most of these studies focused on examining the hyperelastic mechanical properties of rubber materials at room temperature and in large deformation range (strain exceeding 300%) and did not discuss the selection of models for rubber experimental data at different temperatures. Although most of the rubber components are in the medium deformation range (strain less than 150%), there are relatively few studies on the temperature dependence of the mechanical behavior of filled rubber in the medium deformation range.

In summary, there are few systematic studies on the mechanical properties and temperature dependence of filled rubber, which is difficult to meet the production and design requirements at this stage. Therefore, in order to study the mechanical properties of rubber more accurately, it is necessary to conduct the experimental tests on the temperature-dependent mechanical properties of the filled rubber in a larger deformation range, and to investigate the ability of various rubber constitutive models to characterize the experimental data. It is very important to find or establish a constitutive model that can accurately describe the mechanical characteristics of filled rubber under various deformation states, and to study the ability of the model to describe the temperature dependence of the rubber compound in the medium deformation range.

The main purpose of this paper is to explore the law of hyperelastic temperature dependence of filled rubber under certain deformation, investigate the ability of constitutive models to characterize the temperature dependence of rubber hyperelasticity, reveal the internal laws of temperature dependence of rubber mechanical properties through material parameters, propose a more accurate constitutive model, and study the ability of constitutive models to predict the stress–strain behavior of different deformation states. 

## 2. Hyperelastic Constitutive Model of Rubber Materials

According to the different theoretical foundations, the constitutive models of hyperelastic materials are mainly divided into two categories, i.e., the phenomenological model based on the continuum mechanics theory, and the molecular chain network model based on the statistical mechanics theory. This section will briefly overview the representative hyperelastic constitutive models of rubber materials.

### 2.1. Invariant-Based Continuum Mechanics Treatments

Rivlin proposed the most general expression of the strain energy density in the form of invariant series [8].

(1) $$W\left(I_{1},I_{2}\right)=\overset{n}{\underset{i,j=0}{\sum }}C_{ij}{\left(I_{1}-3\right)}^{i}{\left(I_{2}-3\right)}^{j}$$

where $C_{ij}$ is the Rivlin coefficient, which is the regression coefficient of experimental data and has no specific physical meaning. $I_{1}$ and $I_{2}$ are the first and second strain invariants of the Green strain tensor, respectively.

#### 2.1.1. Neo-Hookean Model

When Equation (1) retains only the first term, Equation (1) becomes Equation (2)

(2) $$W_{NH}=C_{10}\left(I_{1}-3\right)$$

which is often called the neo-Hookean model [9]. This is the simplest hyperelastic constitutive model. In the neo-Hookean model, the coefficient $C_{10}$ is equal to half of the shear modulus, so the neo-Hookean model is unconditionally stable.

#### 2.1.2. Mooney–Rivlin Model

By further simplifying the Rivlin model and setting *n* = 1, the equation first proposed by Mooney [10] is obtained:

(3) $$W_{MR}=C_{10}\left(I_{1}-3\right)+C_{01}\left(I_{2}-3\right)$$

this model is called Mooney–Rivlin model, and it is widely used in the finite element analysis of elastomer deformation. In Equation (3), $C_{10}$ and $C_{01}$ are material positive definite constants. As long as $C_{10}+C_{01}>0$, Mooney–Rivlin model is stable.

#### 2.1.3. Yeoh Model

The analysis of the test data of the carbon black filled rubber compound by Yeoh showed that $\frac{\partial W}{\partial I_{2}}\ll \frac{\partial W}{\partial I_{1}}$, and $\frac{\partial W}{\partial I_{2}}$ was close to zero [11]. The incompressible Yeoh model assumes the strain energy density is a general polynomial function of only the first principal stretch invariant $I_{1}$, as shown in the following equation as Equation (4):

(4) $$W_{Y}=C_{10}\left(I_{1}-3\right)+C_{20}{\left(I_{1}-3\right)}^{2}+C_{30}{\left(I_{1}-3\right)}^{3}$$

where $C_{10}$, $C_{20}$, $C_{30}$ are undetermined material model parameters, not based on physics, and can be determined by uniaxial tensile test.

#### 2.1.4. Reduced Polynomial Model

In Equation (1), when $\;j=0,\;C_{ij}\neq 0$, the reduced polynomial model can be obtained, as shown in the following equation:

(5) $$W=\overset{n}{\underset{i=1}{\sum }}C_{i0}{\left(I_{1}-3\right)}^{i}$$

Neo-Hookean model and Yeoh model are essentially reduced polynomial models with n=1 and n=3, respectively.

### 2.2. Stretch-Based Continuum Mechanics Treatments

Ogden directly used the principal stretch ratio $\lambda_{i}$ as the independent variable to express the strain energy density function in the form of series for incompressible materials [12], which can be expressed by the following equation.

(6) $$W=\overset{n}{\underset{i=1}{\sum }}\frac{u_{i}}{\alpha_{i}}\left(\lambda_{1}^{\alpha_{i}}+\lambda_{2}^{\alpha_{i}}+\lambda_{3}^{\alpha_{i}}-3\right)$$

where $u_{i}$ and $\alpha_{i}$ are arbitrary constants (can be non-integers). When transformed into the form of stress and stretch, the Ogden model is more convenient than the strain energy function expressed by invariants, but the parameters are empirical and lack physical meaning.

### 2.3. Statistical Mechanics Treatments

#### 2.3.1. Gaussian Statistics Model

The first statistical mechanics method to describe the force acting on a deforming polymeric network assumes that Gaussian statistics is applied. This deformation makes the chain length r not approach its fully extended length $nl\left(r\ll nl\right)$, where n is the number of statistical links of length l in the chain between chemical crosslinks. Then the elastic strain energy function, $W_{G}$, can be derived from the change in configurational entropy, as expressed in the following equation:

(7) $$W_{G}=\frac{1}{2}Nk\theta \left(\lambda_{1}^{2}+\lambda_{2}^{2}+\lambda_{3}^{2}-3\right)$$

where N is chain density, k is Boltzmann’s constant, θ is absolute temperature, and $\;\lambda_{i}(i=1,2,3$*)* is the principal stretches. In particular, when $C_{10}=\frac{1}{2}Nk\theta$, Equation (2) is equal to Equation (7) for Gaussian model.

#### 2.3.2. Non-Gaussian Statistics Model

##### Three-Chain Network Model

The earliest non-Gaussian chain network model is the three-chain network model proposed by James and Guth in 1943 [13]. The strain energy function can be expressed as

(8) $$W_{3ch}=\frac{Nk\theta }{3}\sqrt{n}\left[\lambda_{1}\beta_{1}+\sqrt{n}ln\left(\frac{\beta_{1}}{\sinh\beta_{1}}\right)+\lambda_{2}\beta_{2}+\sqrt{n}ln\left(\frac{\beta_{2}}{\sinh\beta_{2}}\right)+\lambda_{3}\beta_{3}+\sqrt{n}ln\left(\frac{\beta_{3}}{\sinh\beta_{3}}\right)\right]$$

where $\beta_{i}=L^{-1}\left(\frac{\lambda_{i}}{\sqrt{n}}\right);\mathrm{for}\;i=1,2,3$, and $L^{-1}\left(x\right)$ is the inverse function of Langevin function $L\left(x\right)=cothx-1/x$.

##### Eight-Chain Model

The eight-chain model based on Langevin statistical theory proposed by Arruda and Boyce (1993) is a relatively successful non-Gaussian chain network model [14]. The strain energy density function of the Arruda-Boyce model is

(9) $$W_{8ch}=Nk\theta \sqrt{n}\left[\beta_{c}\lambda_{c}+\sqrt{n}ln\left(\frac{\beta_{c}}{\sinh\beta_{c}}\right)\right]$$

where $\lambda_{c}$ is the chain stretch $\lambda_{c}=\frac{1}{\sqrt{3}}I_{1}^{1/2}$, $\beta_{c}$ is the inverse Langevin function $\beta_{c}=L^{-1}\left[\lambda_{c}/\sqrt{n}\right]$. The parameter n is the number of the rigid segments in a single chain, which is a temperature-independent constant for the pure rubber compound without fillers. However, n is indeed temperature-dependent for the reinforced rubber compound due to the steric hindrance effect of carbon black particles on the rubber molecular chain [15,16,17].

### 2.4. A New Improved Eight-Chain Model

In this paper, in order to improve the accuracy and predictive ability of the model and to make the calculation and fitting process more convenient and accurate, combining the advantages of the eight-chain model and the constitutive model of vascular hyperelastic materials [14,18,19,20], a new improved eight-chain model was proposed by introducing the eight-chain model into the exponential correction term. Two additional parameters were introduced in the correction term, i.e., the global interaction factors a and b, which can characterize the interaction effect between the components inside the filled rubber. The exponential strain energy density has been proved to have the comparatively high ability to reflect the mechanical behavior [9,21,22,23,24]. Based on the relationship between the Helmholtz free energy change and the total entropy change of the chain per unit volume, the strain energy density function of the new model can be obtained as follows:

(10) $$W_{new}=\mu \left\{\sqrt{n}\lambda_{c}\beta_{c}+n\ln\left(\frac{\beta_{c}}{\sinh\beta_{c}}\right)+\frac{a}{b}\left[e^{b\left(I_{1}-3\right)}-1\right]\right\}$$

Four material parameters, μ, n, a and b, need to be determined in the improved eight-chain model. Combining with the theory in the publication by Rivlin and Treloar [25,26,27], the series expansion relationship of stress–stretch in the uniaxial extension of the improved eight-chain model can be obtained as follows:

(11) $$\sigma =2\mu \left(\lambda -\lambda^{-2}\right)\left[\overset{5}{\underset{i=1}{\sum }}\frac{iC_{i}}{\;n^{i-1}}I_{1}{}^{i-1}+ae^{b\left(I_{1}-3\right)}\right]$$

where $C_{1}=\frac{1}{2},C_{2}=\frac{1}{20},C_{3}=\frac{11}{1050},C_{4}=\frac{19}{7000},C_{5}=\frac{519}{673750}$, and μ=Nkθ.

## 3. Experiments

### 3.1. Experimental Materials

Two types of rubber materials filled with different contents of carbon black were used in the experiments. The codes and formulas of the two types of rubber used in the tests are shown in Table 1. Natural rubber was obtained from Shandong Fushun New Material Technology Co., Ltd., Jinan, Shandong Province, China. CB was obtained from Qingdao Jinlan Chemical Co., Ltd., Qingdao, Shandong Province, China. Other agents were commercially available. Stearic acid, zinc oxide, sulfur, accelerator NS, and antioxidant 4020 were industrial grade products.

### 3.2. Sample Preparation

The rubber mixing was divided into two stages. In the first mixing stage, raw rubber, zinc oxide, stearic acid, and carbon black were added into an internal mixer and mixed evenly. In the second mixing stage, sulfur and accelerator were added to the mixture from the first stage into an open roll mixing mill and mixed evenly. After pressing the mixture into thin slices, let them stand for less than 2 h. The vulcanizations were conducted at 150 °C under 10 MPa, and the curing time was the optimum cure time (T_90_).

The stretching specimen was prepared according to ISO 37-2017 (dumb-bell Type 2). The fixture of uniaxial tensile test at different temperatures used a double eccentric wheel clamp, RA-4-1, which was a special tensile clamp for rubber. Specifically, to ensure the specimen reached the required test temperature, the temperature control box was stabilized for 10 min after reaching the test temperature before the tensile test. In order to eliminate the Mullins effect of the rubber material [28], each sample underwent 10 loading-unloading cycles at a speed of 100 mm/min. The cyclic strain was 150%, and the temperature was 291 K. The modulated specimens were placed at room temperature for 24 h before uniaxial tensile test. The experiment was repeated at least 5 times under each condition, and the obtained average value was used as the final experimental result.

## 4. Experiment Results

The deformation of rubber is generally less than 100% in terms of engineering strain, and 150% strain can be applied to most working conditions. Thus, in the uniaxial tensile test, 150% strain was used to characterize the mechanical properties of rubber materials.

In this section, the nominal stress–strain curve and the Mooney curve were used to study the nonlinear characteristics of the mechanical behavior of rubber compound at different temperatures. The horizontal axis of Mooney curve is the reciprocal of the stretch ratio, i.e., 1/λ, and the vertical axis is “reduced stress $\;f_{\mathrm{red}}=f/\left(\lambda_{1}-\lambda_{1}^{-2}\right)$”, where f is the corresponding nominal stress [29].

**Figure 1:**
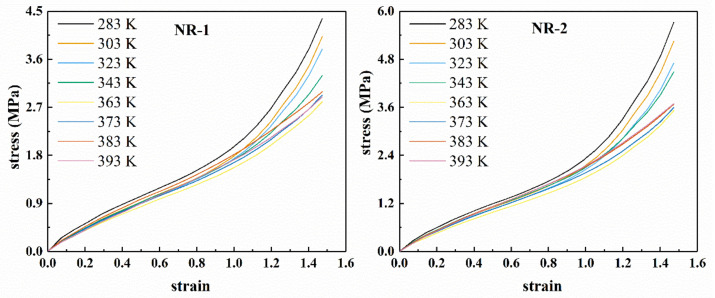
Uniaxial tensile stress–strain curves of rubber compounds NR-1 and NR-2 at different temperatures.

**Figure 2:**
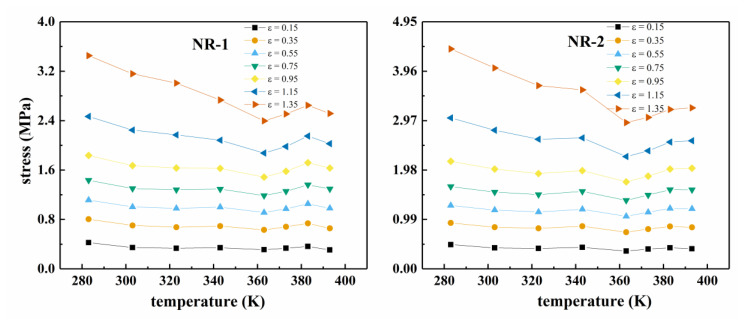
Stress–temperature curves at different constant strains for NR-1 and NR-2 (*ε* is the value of the nominal strain).

**Figure 3:**
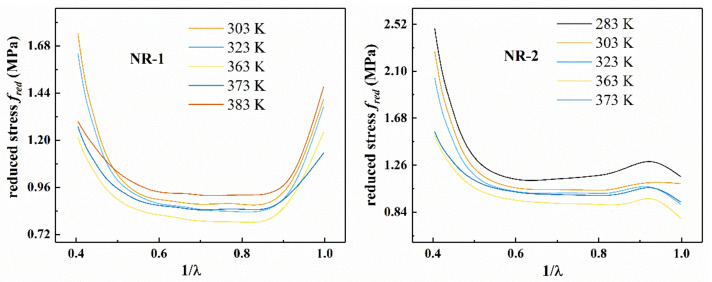
Mooney plots of NR-1 and NR-2 under uniaxial tension at different temperatures.

Figure 1 shows the uniaxial tensile nominal stress–strain curves of two types of CB filled rubber at different temperatures. Figure 3 shows the corresponding Mooney curves. Due to the difference in the properties of the two kinds of filled rubber, the temperature range of the Mooney curve varies with the rubber compound. Figure 2 shows the relationship between the stress and temperature of the two types of rubber under constant strains. From Figure 1, it can be seen that all the nominal stress–strain curves of filled rubber at different temperatures exhibit obvious “S-shape” non-linear characteristics, that is, the mechanical behavior of filled rubber still maintains highly monotonic nonlinearity, but the non-linear degree of the stress–strain curves does not change with the temperature. According to Figure 1 and Figure 2, unlike the natural vulcanized rubber without carbon black filling [29,30,31,32], under constant strain, the stress of carbon black filled rubber varies nonlinearly with the increase of temperature. When the temperature increased, the overall stress response of the filled compound changed with temperature in a more complex pattern. In the range of 283 K–363 K, the overall stress–strain curve of the rubber decreased with the increase of temperature. Thus, in this temperature range, the rubber material became softer with the temperature. When the temperature exceeded 363 K, the overall stress–strain curve of the rubber compound was higher than the 363 K’s, that is, with the increase of temperature, the modulus stiffness of the rubber compound increased and the rubber became harder. Combined with Figure 1 and Figure 2, the variation of stress is relatively disorderly around 393 K, and the stress at constant stretch has a downward tendency again. It appears that at constant stretch, the stress at 393 K is lower than that at 383 K. In the entire temperature range, the constant elongation stress showed a highly nonlinear change with temperature. Considering that the filling of carbon black particles aggravated the viscoelastic response of the rubber compound [33,34,35], the nonlinearity of the stress-temperature relationship of the filled rubber was much more severe than that of the natural vulcanized rubber.

According to Figure 3, temperature has significant effect on the reduced stress of filled rubber. From Figure 3, as the temperature increases, the initial modulus of the rubber first decreases, and then increases after the temperature reaches 363 K. The absolute value of the slope and the reduced stress of the Mooney plot increased with the increase of the tensile ratio λ at larger stretches（$\frac{1}{\lambda }<0.55$）, which suggested that the deviation from Gaussian behavior was due to the interactions or constraints of neighboring chains. In addition, the “up-turn” indicated the non-Gaussian regime of chain stretch. The Mooney plot of NR-1 showed an overall upward trend at $0.85<\frac{1}{\lambda }$, and the reduced stress gradually decreased with the increase of the tensile ratio λ, which supported the Mooney–Rivlin model [2]. However, NR-2 showed a downward trend at $\;0.9<\frac{1}{\lambda }$. At $0.55<\frac{1}{\lambda }<0.85$, the Mooney plot showed a straight line with a slope of zero, which indicated that the material obeyed Gaussian statistics.

In conclusion, because the interaction between carbon black particles and rubber matrix was obviously affected by temperature, the filled rubber exhibited a more complex temperature-dependent mechanical behavior than pure rubber.

## 5. Analysis and Evaluation of the Ability of Some Hyperelastic Models to Characterize Temperature Dependence

**Figure 4:**
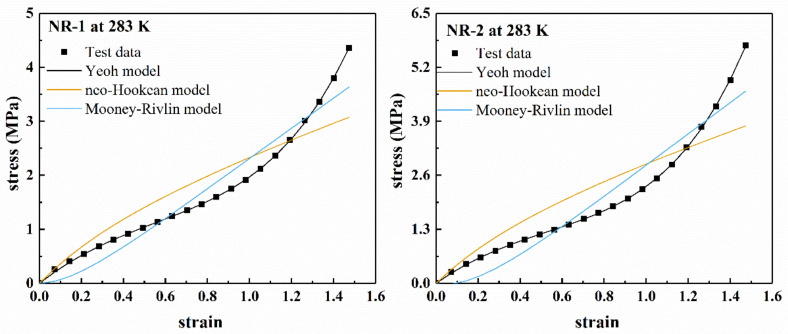
Fitting results of polynomial model for filled rubber NR-1 and NR-2 under uniaxial tension at 283 K.

**Figure 5:**
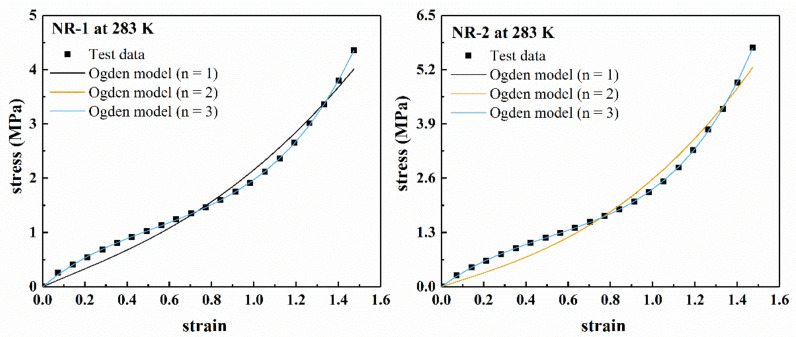
Fitting results of Ogden model for filled rubber NR-1 and NR-2 under uniaxial tension at 283 K.

Figure 4 shows the fitting results of several polynomial models for the uniaxial stretching of rubber materials NR-1 and NR-2 at 283 K in a larger deformation range (nominal strain *ε* < 1.5). Figure 5 shows the fitting results of Ogden model for uniaxial tension. It can be seen from Figure 4 that the neo-Hookean model and Mooney–Rivlin model cannot fit the “S-shape” characteristics of the stress–strain curve under larger deformation, and there are obvious deviations between the fitting results and the uniaxial tensile test data. Therefore, the neo-Hookean model and Mooney–Rivlin model cannot accurately characterize the nonlinear change of material stiffness under larger deformation. The Yeoh model has better fitting results.

It can be found from Figure 5 that for the experimental data of NR-1 at 283 K, when *n* = 1, the Ogden model has poor fitting results. When *n* = 2 and *n* = 3, the fitting curves overlap, and the model is in good agreement with the experimental data. For NR-2 at 283 K, when *n* = 1 and *n* = 2, the fitting curves of Ogden model overlap. Only when *n* = 3, the fitting curve can well describe the experimental data. The results show that when *n* = 2, the ability of the model to describe experimental data was unstable.

Therefore, the fitting ability of Ogden model to data increased with the increase of the value of *n*. However, the increase of *n* led to the increase of model parameters, which increased the complexity of the model. For the test materials in this paper, *n* = 3 was the most appropriate.

In order to evaluate the goodness of fit of the eight-chain model and the improved eight-chain model, we calculated the Total Sum of Squares (TSS) and Residual Sum of Squares (RSS), and obtained the coefficient of determination $R^{2}$.

(12) $$\begin{array}{c} TSS=\sum_{i=1}^{N}{\left(P_{i}-\bar{P}\right)}^{2} \end{array}$$

(13) $$\begin{array}{c} \begin{array}{c} \begin{array}{c} RSS=\sum_{i=1}^{N}{\left({\hat{P}}_{i}-P_{i}\right)}^{2} \end{array} \end{array} \end{array}$$

(14) $$R^{2}=1-RSS/TSS$$

where $P_{i}$ is the experimental value, $\bar{P}$ is the average value of $P_{i}$, ${\hat{P}}_{i}$ is the model fitting value, and N is the number of experimental data points participating in the fitting. The larger the $R^{2}$, the higher the overall goodness of the model.

**Figure 6:**
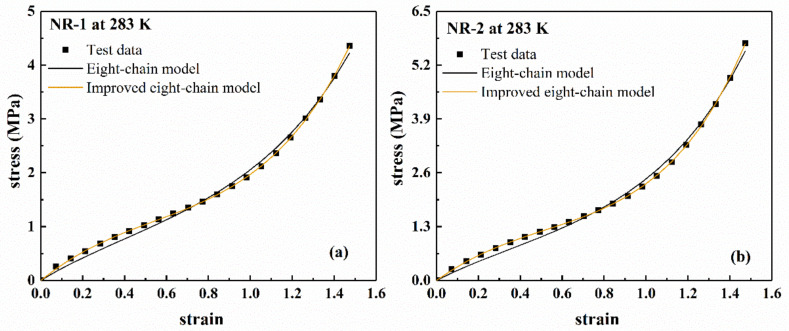
Fitting results of eight-chain model and improved eight-chain model for filled rubber under uniaxial tension, (**a**) NR-1; (**b**) NR-2.

**Figure 7:**
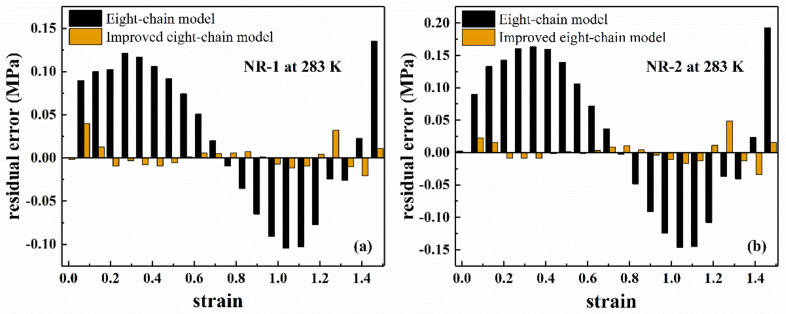
The residual error of fitting results of eight-chain model and improved eight-chain model for filled rubber under uniaxial tension, (**a**) NR-1; (**b**) NR-2.

Figure 6 shows the fitting results of the eight-chain model and the improved eight-chain model. The residuals of the fitting results are shown in Figure 7. The values of $R^{2}$ of both models are listed in Table 2. From Table 2, it can be seen that the improved eight-chain model provides a better fit for both types of rubber than the eight-chain model. From Figure 6 and Figure 7, it can be seen that although the eight-chain model can reflect the main characteristics of the mechanical behavior of the rubber to a certain extent, there is still a significant deviation between the fitting results and the experimental data. In the entire deformation range, the fitting result of the improved eight-chain model is better than that of the eight-chain model. In summary, the improved eight-chain model has a better fitting effect.

In summary, Yeoh model, Ogden model (*n* = 3), and the improved eight-chain model are excellent in describing the data. Subsequently, the following sections will focus on evaluating the capability of Yeoh model, Ogden model (*n* = 3) and improved eight-chain model to characterize the temperature dependence.

**Figure 8:**
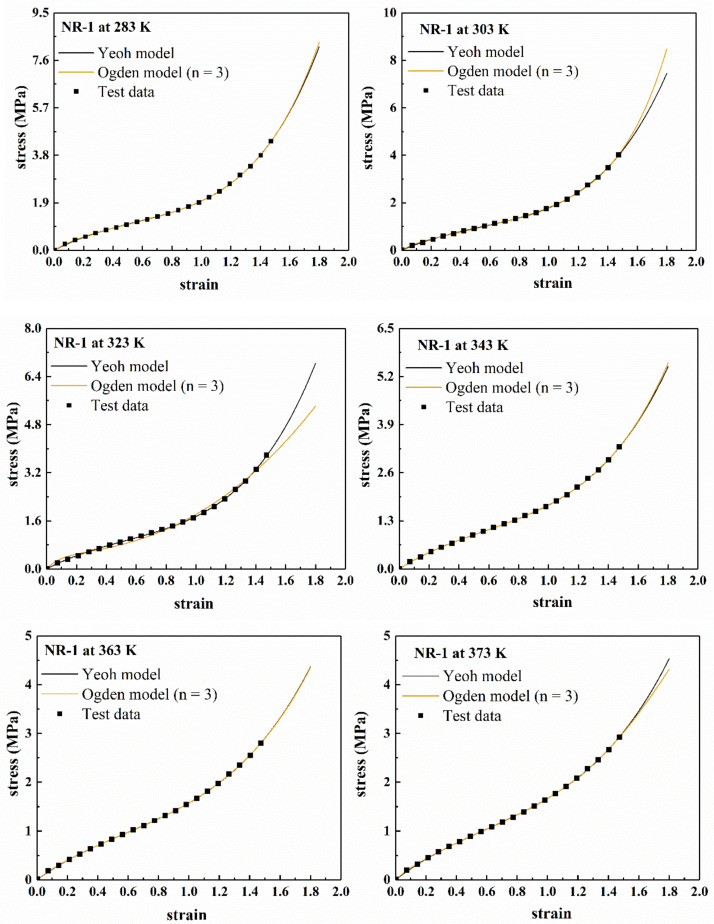

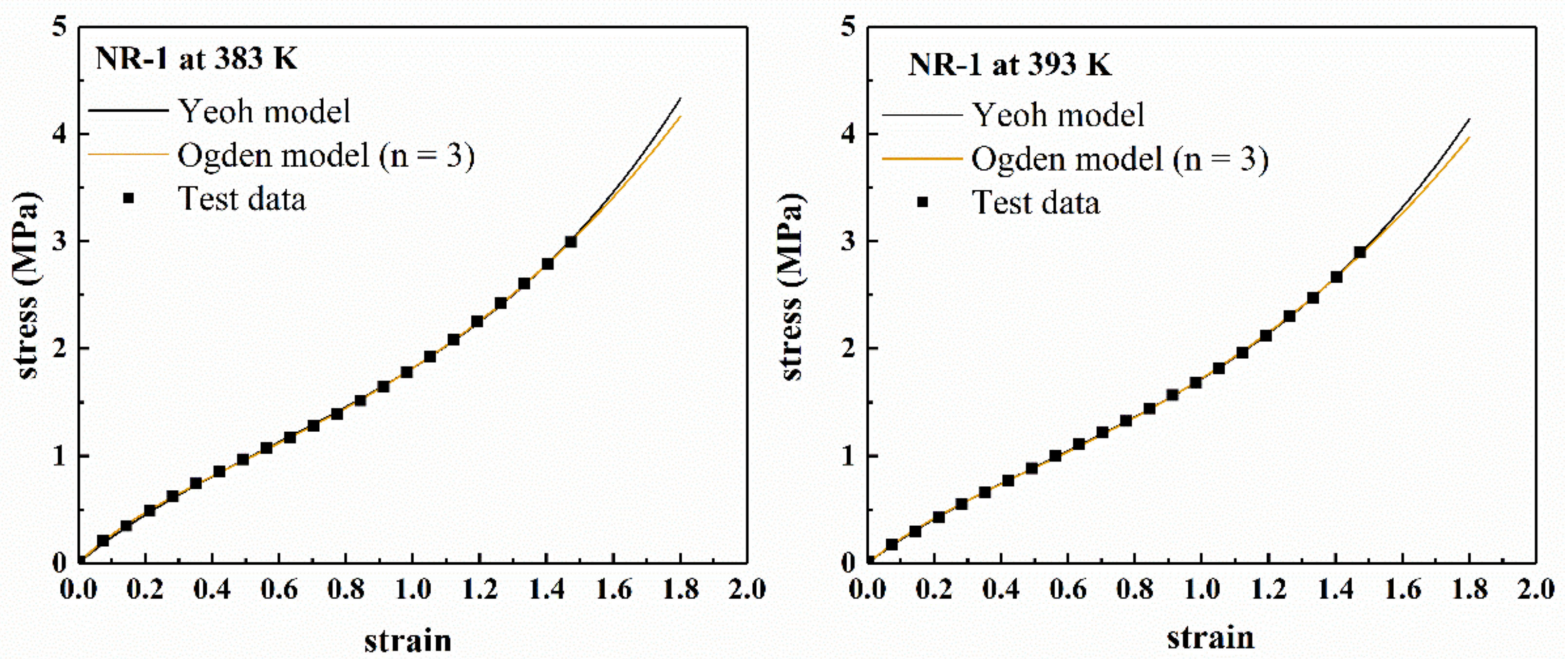
The fitting results of the Yeoh model and the Ogden model (*n* = 3) for NR-1 under uniaxial tension at different temperatures.

It can be seen from Figure 8 that within the range of experimental data (*ε* < 1.5), there is almost no difference between the fitting curves of the two models, which demonstrates that both models can reasonably describe the experimental data within the range. In addition, the “S shape” of the experimental curve is also well characterized in the fitting curves. For the prediction curves beyond the range of experimental data (*ε* > 1.5), the two models only have large differences at 303 K and 323 K, but the shapes of the prediction curves are roughly the same. Thus, it is impossible to judge the pros and cons of the prediction curves of the two models. At other temperatures, the prediction curves of the two models are basically the same. However, using Ogden model (*n* = 3), the parameters of the model were extremely difficult to converge, and the calculation time was long, which may cause inconvenience in the application of the model.

The fitting parameters of Yeoh model for NR-1 and NR-2 experimental data at different temperatures are listed in Table 3. The following relationship was obtained by fitting the experimental data.

(15) $$\left\{\begin{array}{c} C_{10}\\ C_{20}\\ C_{30} \end{array}\right\}=\left(\begin{array}{ccc} C_{10}{}^{1} & C_{10}{}^{2} & C_{10}{}^{3}\\ C_{20}{}^{1} & C_{20}{}^{2} & C_{20}{}^{3}\\ C_{30}{}^{1} & C_{30}{}^{2} & C_{30}{}^{3} \end{array}\right)\left\{\begin{array}{c} 1\\ T\\ T^{2} \end{array}\right\}$$

**Figure 9:**
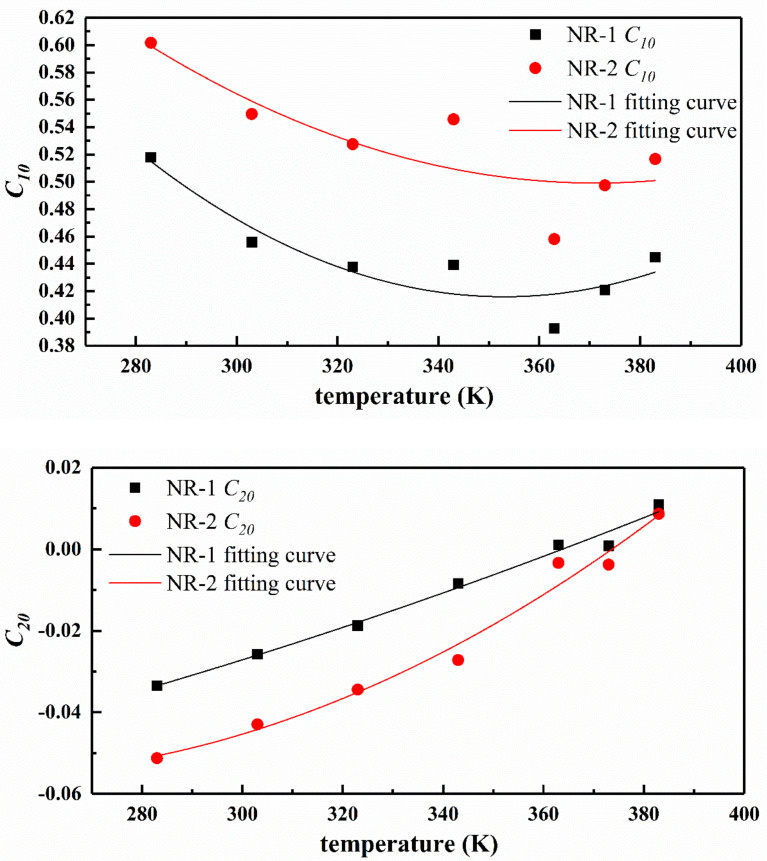

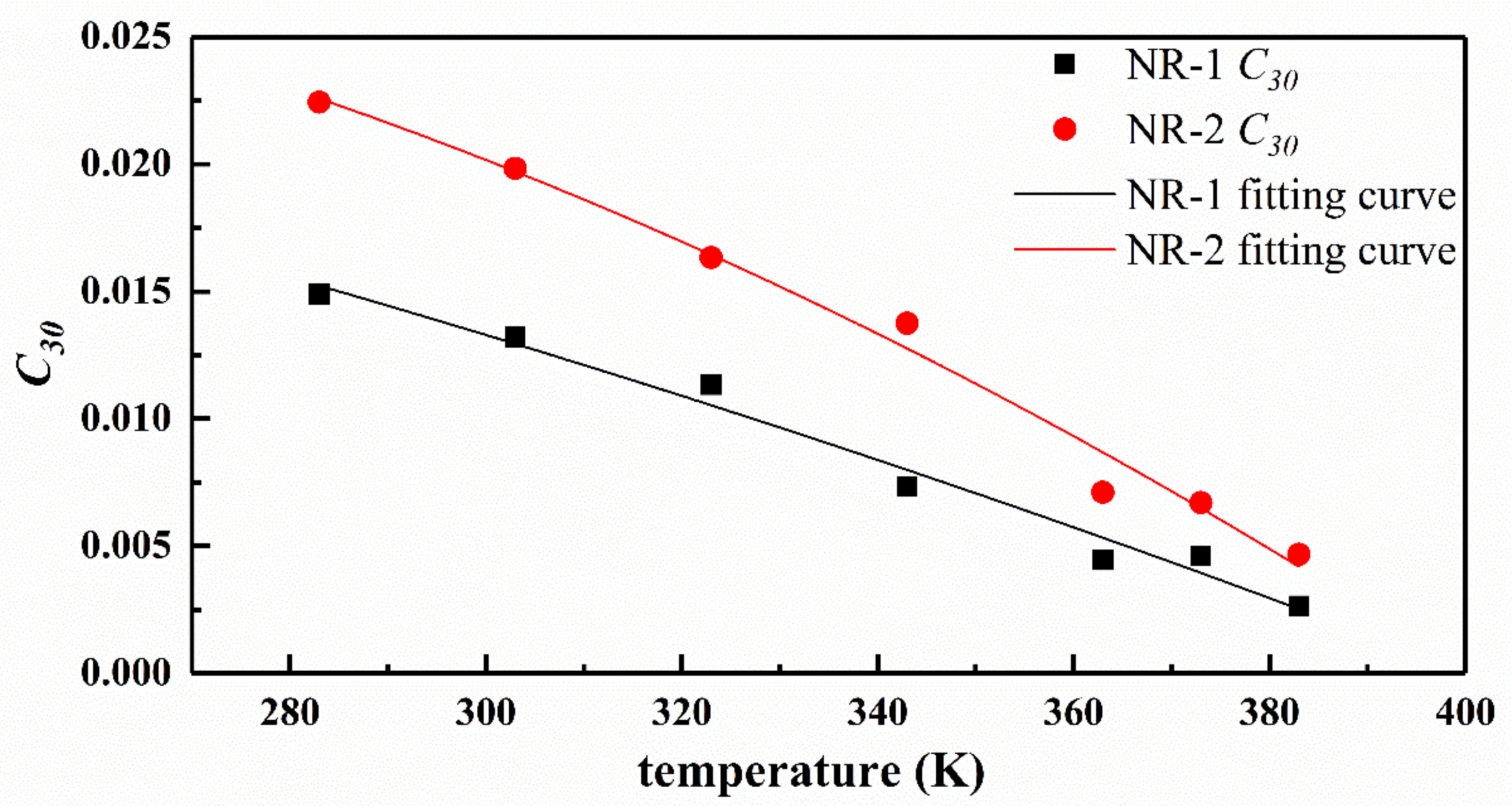
Trend of Yeoh model parameters with temperature and the corresponding quadratic fitting curves.

**Figure 10:**
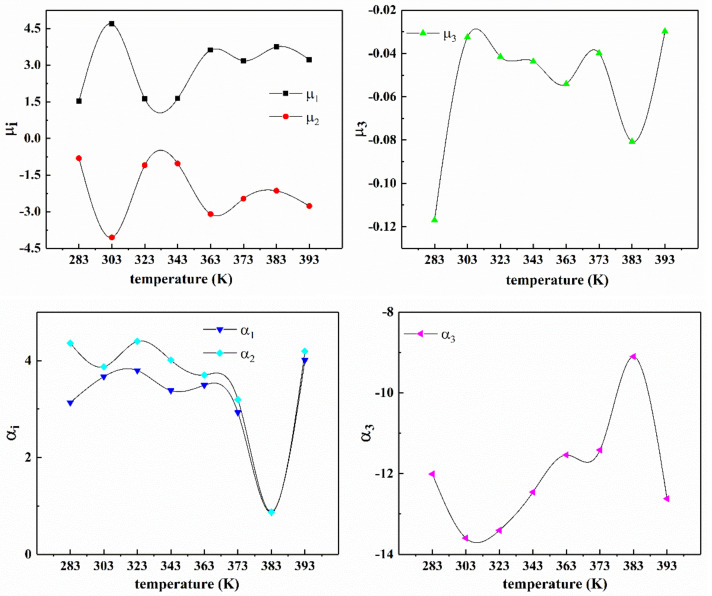
Trend of the Ogden model (*n* = 3) parameters with temperature obtained by fitting the uniaxial tension data of filled rubber NR-1 at different temperatures.

Figure 9 shows the relationship of Yeoh model parameters with temperature and the fitting curve of parameters according to Equation (15). As shown in Figure 9, the trend of Yeoh model parameter $C_{10}$ with temperature is similar to the stress-temperature curve, that is, as the temperature increases, the parameter value first decreases and then increases, and the inflection point is between 353 K and 373 K. In conclusion, $C_{10}$ varies approximately as a quadratic function with temperature, and has a certain temperature dependence. The parameters $C_{20}$ and $C_{30}$ almost change linearly with temperature. Figure 10 and Table 4 show the relationship between the parameters of Ogden model (*n* = 3) and temperature. As can be seen from the diagram, the parameters of Ogden model have no law with the change of temperature, so it can be said that the parameters have no temperature dependence.

In summary, Yeoh model and Ogden model (*n* = 3) have good ability to describe the experimental results at different temperatures and to predict beyond the range of experimental data. However, as mentioned earlier, the Yeoh and Ogden model parameters are empirical and lack physical meaning. So, it is difficult to ensure that the two models can correctly describe the nonlinear characteristics of rubber under various deformation states only by using the model parameters determined by a single deformation form. Moreover, the Ogden model (*n* = 3) has too many model parameters and poor fitting convergence.

**Figure 11:**
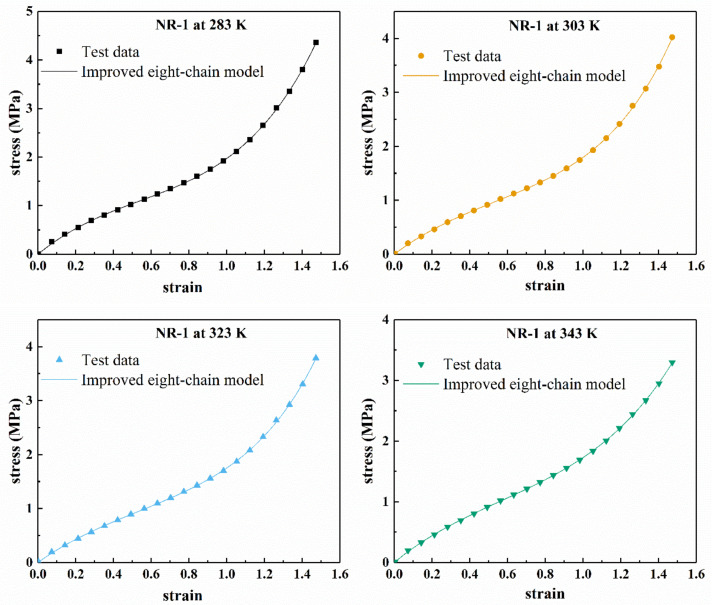

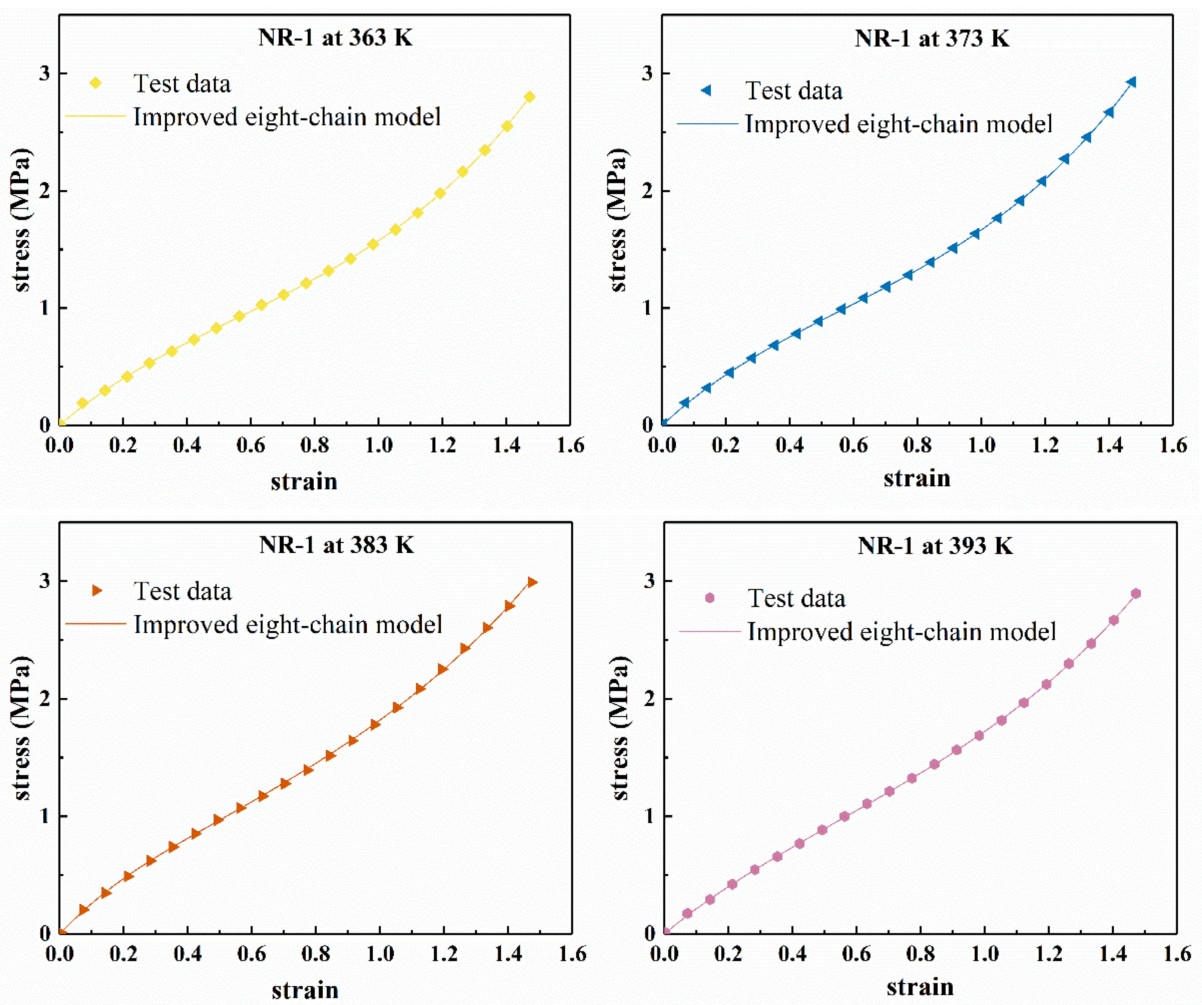
Fitting results of NR-1 under uniaxial tension at different temperatures by improved eight-chain model.

**Figure 12:**
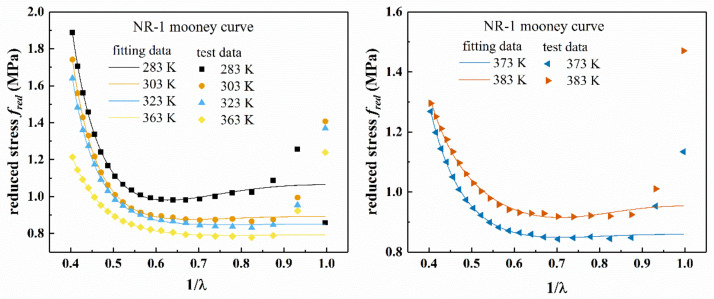
Mooney curves by improved eight-chain model for uniaxial tension of NR-1 at different temperatures.

Figure 11 shows the fitting results of the improved eight-chain model for the uniaxial tension of the filled rubber NR-1, and Figure 12 shows the Mooney curve of the model. From Figure 11, it can be seen that the improved eight-chain model can accurately describe the uniaxial tensile mechanical behavior of the rubber NR-1 at different temperatures and can reasonably reflect the “S shape” of stress–strain curve of the filled rubber in the studied deformation range. Figure 12 shows that when $\frac{1}{\lambda }<0.9$, the improved eight-chain model can reasonably describe the Mooney curve of the rubber at different temperatures; however, when $\frac{1}{\lambda }>0.9$, there is a larger difference between the fitting curve of the improved eight-chain model and the Mooney curve, and the reduced stress of the test data is obviously larger than the fitting value. This phenomenon is due to the fact that in the early stage of tension, the stress is relatively small, and the equipment error is relatively large. Therefore, in the early stage of tension, the test stress was larger than the actual stress, and the improved eight-chain model corrected the equipment error to a certain extent. After model correction, the curve in Figure 12 shows linear behavior in the small to moderate stretch range, and an upward trend in a larger stretch range.

**Figure 13:**
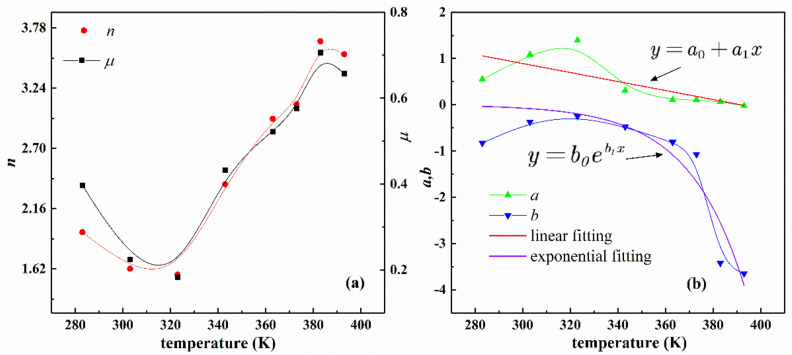
Temperature-dependent curves of fitting parameters of NR-1 under uniaxial tension at different temperatures by improved eight-chain model. (**a**) Curves of parameters n, μ with temperature; (**b**) Curves of parameters a, b with temperature and the corresponding fitting curves.

Table 5 lists the values of the fitting parameters of the improved eight-chain model according to the test data of the filling rubber NR-1 at different temperatures. Figure 13 shows the variation trend of the four material parameters of the improved eight-chain model with temperature. From Figure 13a, the changes of parameters μ and n are approximately a quadratic function of temperature, and the shape and trend of the curves of the two parameters are approximately the same, which shows that the physical properties of the rubber compound also vary regularly with temperature. According to Figure 13b, it is found that the parameter a changes approximately linearly with temperature, and the parameter b changes exponentially with temperature. It can be concluded that the parameters of the improved eight-chain model have the law of temperature dependence.

## 6. Analysis and Evaluation of the Predictive Ability of the Hyperelastic Model in Various Deformation States

If only uniaxial tension data is used, a set of coefficients can be obtained through each constitutive model. After the coefficients are determined, the constitutive model can predict biaxial tension and pure shear curves. According to the conclusions of the previous section, the Yeoh model and the improved eight-chain model were selected to evaluate the predictive ability of the experimental data.

**Figure 14:**
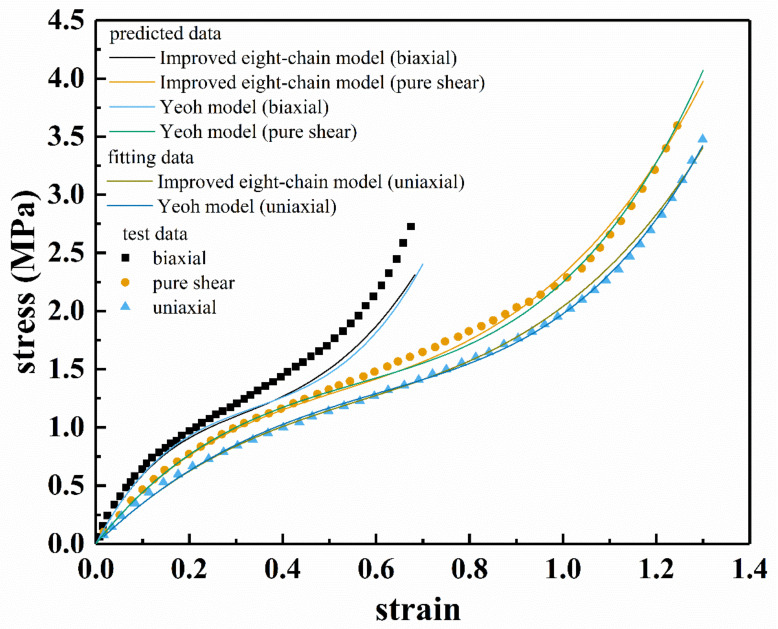
The prediction results of the Yeoh model and the improved eight-chain model for pure shear and biaxial tension according to the fitting parameters of uniaxial tension.

Based on the data in Reference [36], the fitting results of the improved eight-chain model and the Yeoh model for uniaxial stretching of a certain filled rubber material as well as the prediction results under pure shear and biaxial tension deformation were obtained, as shown in Figure 14. Table 6 lists the model fitting parameters and correlation coefficient *R* corresponding to uniaxial tension. It can be seen from Figure 14 and Table 6 that both models can well characterize the stress–strain relationship under uniaxial tension. In addition, when the pure shear (*ε* < 0.2) and biaxial tension (*ε* < 0.6) are in a small deformation range, the predictions of the two models are very accurate. However, under a larger deformation, the prediction results of the two models for pure shear (*ε* > 0.6) and biaxial tension (*ε* > 0.2) have deviation from the test results. However, in a larger deformation range, the improved eight-chain model still has better predictive ability than the Yeoh model.

**Figure 15:**
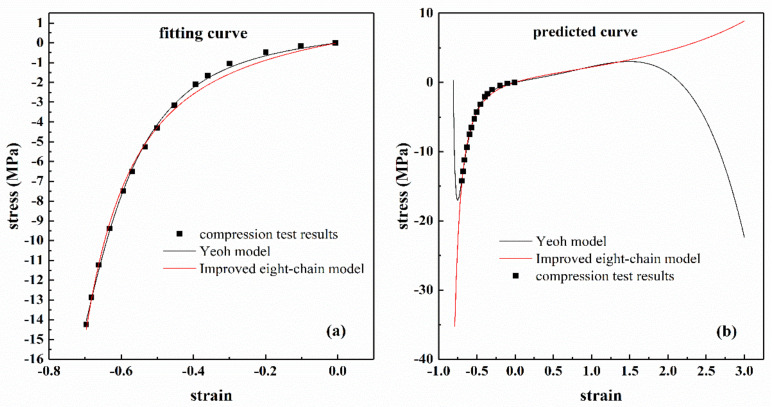
Nominal stress versus strain on Przybylo and Arruda data in uniaxial compression and characterization results for the Yeoh model and the improved eight-model. (**a**) Fitting results of uniaxial compression data; (**b**) extended compression range and predicted tension showing the instability of the Yeoh model and the improved eight-model.

Figure 15a shows the stress–strain curve on the Przybylo and Arruda compression test results [37], and the Yeoh and improved eight-chain models were used to fit the experimental data. The fitting results are also shown in Figure 15a. The corresponding fitting coefficients and correlation coefficients *R* are listed in Table 7. From Figure 15a and Table 7, it can be found that both models can accurately reproduce the uniaxial compression data. When the uniaxial compression analysis of each model was extended beyond the characteristic range, the Yeoh model failed to predict the expected response, as shown in Figure 15b, which was due to the fact that as the compressive stretch increased, the nominal stress had a smaller and smaller negative value. The constants in Table 7 were used to predict uniaxial stretching, and the results are shown in Figure 15b. The predicted tensile results were similar under moderate stretches (*ε* < 1.5) for both models, but varied substantially under large stretches (*ε* > 1.5), only the improved eight-chain model can reasonably predict the actual physical response of tension, the Yeoh model become unstable in tension and predict unrealistic responses. This result was due to the fact that the Drucker’s stability of the Yeoh model was not considered [38]. However, considering the stability will sacrifice the fitting accuracy of the model. The improved eight-chain model is extended from the eight-chain model, so the parameters have inherent stability. Therefore, from this perspective, the improved eight-chain model is superior to the Yeoh model.

## 7. Finite Element Simulation

The subroutine UHYPER of the finite element software ABAQUS (Dassault Systèmes, Paris, FRANCE) used in the simulation can customize the material constitutive model for the user, and program the Equation (10), that is, the improved eight-chain model, into the user subroutine. A finite element model of uniaxial tension according to ISO 37-2017 (dumb-bell Type 2) was established in ABAQUS, the thickness of the specimen is 2 mm and then the uniaxial tension of NR-1 at 283 K was simulated using the parameters in Table 5. When performing tensile simulation, the degree of freedom at the left end of the model needs to be completely fixed, and a displacement of about 80 mm is applied to the right end.

**Figure 16:**
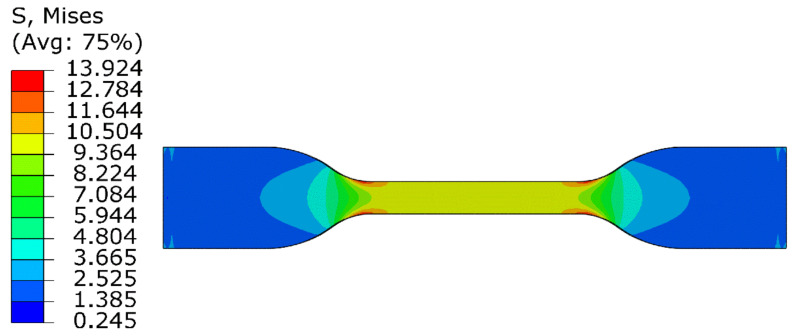
The stress contour profile of rubber specimen NR-1at 283 K by finite element analysis (FEA).

**Figure 17:**
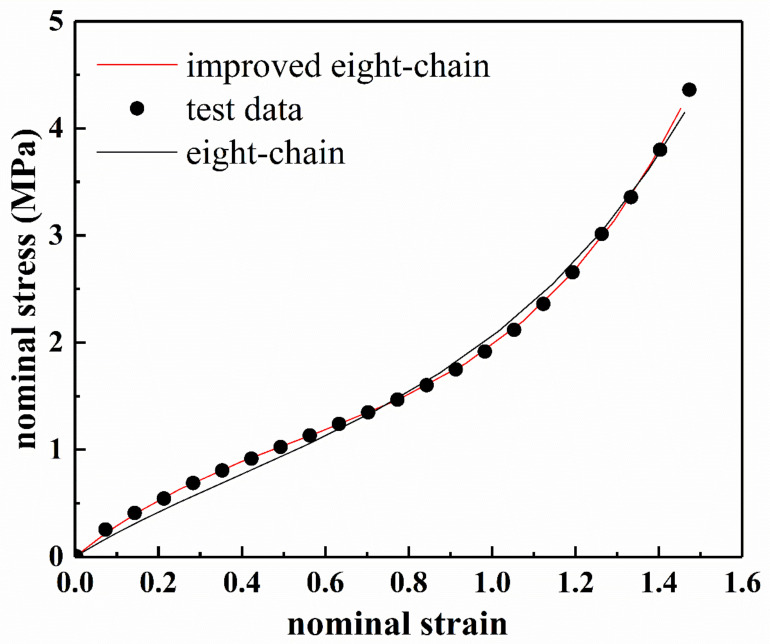
The stress–strain curves of the FEA results and experimental data of the rubber specimen NR-1 at 283 K.

Figure 16 shows the stress contour profile of the improved eight-chain model in uniaxial tension analysis. From the figure, the stress distribution in the middle part is uniform, and the stress concentration appears on the arc where the specimen changes from narrow to wide. Moreover, the stress–strain curve of the tensile was obtained from the uniform deformation region in the middle, as shown in Figure 17. It can be seen from Figure 17 that the simulated tensile data are in good agreement with the experimental data, and the fitting curve of the improved eight-chain model is obviously more accurate than the eight-chain model. Thus, it is obvious that the improved eight-chain model has a better ability to predict the uniaxial tensile test data and can fully ensure the accuracy requirements, thus it can be well applied to the actual working conditions. Moreover, the improved eight-chain model has good engineering practicability.

## 8. Discussion

The improved eight-chain model provides a more convenient and accurate method for the FEA. Since the improved eight-chain model can reasonably predict the experimental data in different deformation states, it is possible to apply the improved eight-chain model to the finite element simulation under multiaxial stress–strain states. The parameters μ and n of the improved eight-chain model inherit the stability of the eight-chain model parameters, and the improved model has higher accuracy, which can be applied to the application scenarios with accuracy requirements, such as accurate prediction of tire sinking and characterization of the mechanical behavior of some precision rubber parts. At the same time, it should be pointed out that the correction term of the improved eight-chain model is semi-empirical, and the specific physical meaning of the internal interaction parameters  a and b is not clear. In this regard, the meaning of the parameters of the eight-chain model is clearer. It will be a very meaningful research direction to discover and explain the physical meaning of the correction parameters a and b.

## 9. Conclusions

(1) The phenomenological model and the molecular chain model of statistical mechanics are theoretically summarized. By combining the advantages of exponential function to describe the hyperelastic behavior of rubber, the eight-chain model was modified, and an improved eight-chain model was proposed.

(2) The temperature-dependent laws of the mechanical behavior of the filled rubber in a larger deformation range were summarized. When the temperature increased in the range of 283 K to 363 K, the uniaxial tensile modulus of the rubber decreased accordingly. When the temperature was higher than 363 K, the uniaxial tensile stress–strain curve of the rubber went up as a whole, the modulus of the rubber compound increased with the increase of temperature, and the rubber compound became hard. At 393 K, the mechanical state of rubber was no longer stable, and the curve had a downward trend, that is, the rubber compound became soft again. Compared with natural rubber, the mechanical properties of filled rubber showed more complex temperature dependence.

(3) Based on the uniaxial tensile data at different temperatures, the ability of several classical hyperelastic constitutive models to describe temperature dependence was evaluated. The results showed that the improved eight-chain model can accurately describe the uniaxial tensile mechanical behavior of two rubber compounds at different temperatures and can reasonably reflect the non-linear changes of the stress–strain curves at different temperatures. The Yeoh and Ogden models also had good ability to describe temperature dependence. However, although the higher-order Ogden model (*n* = 3) had good fitting effect, it contained too many parameters and the fitting convergence was difficult. On the contrary, the low-order Ogden model (*n* = 1, *n* = 2) had poor ability to describe data. The parameters of the Yeoh model are empirical, and if the parameters of the model are only determined by a single deformation state, it is difficult to ensure that the model can correctly describe the nonlinear characteristics of rubber under various deformation states. Compared with the improved eight-chain model, the polynomial model and the eight-chain model had more challenges to accurately reflect the nonlinear elastic mechanical properties of rubber compound at different temperatures.

(4) The improved eight-chain model proposed and developed in this paper can accurately describe the deformation state of the filled rubber in the entire deformation range. Another advantage of the improved eight-chain model is that it is based on the eight-chain model, so its parameters and prediction curves inherit the inherent stability of the eight-chain model. In addition, the improved eight-chain model had high practicality because only one test was required to determine the model parameters of the rubber, and then the mechanical response of rubber under other strain states can be fully characterized by the model.

(5) Finally, the finite element software ABAQUS was used to simulate the uniaxial tension of rubber specimens, and the simulation results were compared with corresponding test data to verify the reliability of material parameters. The results showed that the constitutive model proposed in this paper can effectively characterize the static mechanical properties of carbon black filled rubber and can be easily embedded into the existing finite element software. It has the characteristics of few material parameters, accurate results, strong engineering practicability, etc.

## Figures and Tables

**Table 1:** Ingredient of the two types of carbon black filled rubber (UNIT: PHR).

Ingredient	NR-1	NR-2
NR ^1^	100	100
CB ^2^ N234	40	
CB N330		50
Zinc oxide	5	5
Stearic acid	3	3
Sulfur	2.5	2.5
Accelerator NS	0.7	1
Antioxidant 4020	1	1.5
Total	152.2	163

^1^ NR, natural rubber; ^2^ CB, carbon black.

**Table 2:** The goodness of fit of hyperelastic models based on uniaxial tension data at 283 K.

Code	Eight-Chain Model	Improved Eight-Chain Model
NR-1	0.99497	0.99986
NR-2	0.99433	0.99988

**Table 3:** Fitting parameters of Yeoh model to experimental data of filled rubber at different temperatures.

Temp (K)	NR-1				NR-2			
	$C_{10}$	$C_{20}$	$C_{30}$	RSS ^3^	$C_{10}$	$C_{20}$	$C_{30}$	RSS
283 K	0.51792	−0.03349	0.01489	0.0049	0.60166	−0.05124	0.02244	0.00613
303 K	0.4559	−0.02572	0.01322	0.00422	0.54954	−0.04297	0.01984	0.00821
323 K	0.43785	−0.01874	0.01134	0.00447	0.52758	−0.03439	0.01633	0.00501
343 K	0.43915	−0.00837	0.00732	0.00139	0.54577	−0.02718	0.01375	0.00289
363 K	0.39275	0.00105	0.00446	0.00141	0.45801	−0.00333	0.00711	0.00113
373 K	0.42084	0.000879	0.00461	0.00106	0.49749	−0.00378	0.0067	0.00105
383 K	0.44478	0.01088	0.00264	0.00424	0.51669	0.0087	0.00467	0.00183
393 K	0.40372	0.0156	0.00214	0.00137	0.50142	0.01712	0.00363	0.002

^3^RSS—Residual Sum of Squares.

**Table 4:** Fitting parameters of Ogden model (*n* = 3) to test data of filling rubber NR-1 at different temperatures.

Temp (K)	$\mu_{1}$	$\mu_{2}$	$\mu_{3}$	$\alpha_{1}$	$\alpha_{2}$	$\alpha_{3}$	RSS
283 K	1.52779	−0.81295	−0.117	3.13332	4.3555	−12.01159	0.00278
303 K	4.68786	−4.0549	−0.03249	3.66636	3.87013	−13.59815	0.00299
323 K	1.61986	−1.09774	−0.04148	3.79423	4.39486	−13.40366	0.00247
343 K	1.63296	−1.02974	−0.04365	3.38033	4.0081	−12.45593	0.00116
363 K	3.61752	−3.09701	−0.054	3.49144	3.70022	−11.53832	0.00113
373 K	3.17842	−2.4679	−0.03985	2.92938	3.19636	−11.41498	0.00065
383 K	3.74674	−2.14522	−0.08071	0.87307	0.87325	−9.10137	0.00131
393 K	3.21868	−2.77169	−0.02975	4.01001	4.19083	−12.62049	0.00058

**Table 5:** Fitting parameters of the improved eight-chain model to test data of the filling rubber NR-1 at different temperatures.

Temp (K)	μ	n	a	b	RSS
283 K	0.39672	1.949	0.55205	−0.82525	0.00415
303 K	0.22398	1.62185	1.08616	−0.37105	0.00313
323 K	0.18293	1.5684	1.3939	−0.24883	0.00279
343 K	0.43187	2.37823	0.3056	−0.47432	0.00135
363 K	0.52211	2.96567	0.11002	−0.80604	0.00141
373 K	0.57549	3.09489	0.10651	−1.06821	7.86847 × 10^−4^
383 K	0.70578	3.66047	0.06451	−3.42324	0.00253
393 K	0.6568	3.54368	−0.02237	−3.64862	7.59217 × 10^−4^

**Table 6:** Constants determined by nonlinear regression analysis.

Yeoh	$C_{10}\;$ = 0.64154	$C_{20}\;$ = −0.09902	$C_{30}\;$ = 0.02657		R = 0.99949
improved eight-chain	μ = 0.32794	n = 1.71728	a = 1.0955	b = −0.92158	R = 0.99868

**Table 7:** Constants determined from nonlinear regression analysis.

Yeoh	$C_{10}=0.4056$	$C_{20}=0.0935$	$C_{30}\;$ = –0.01056		R = 0.99952
improved eight-chain	μ=0.39496	n=11.02729	a=0.93007	b=0.05959	R = 0.99796

## Data Availability

The data presented in this study are available on request from the corresponding author.
